# Continuous estimation of respiratory system compliance and airway resistance during pressure-controlled ventilation without end-inspiration occlusion

**DOI:** 10.1186/s12890-024-03061-2

**Published:** 2024-05-20

**Authors:** Yuqing Chen, Yueyang Yuan, Qing Chang, Hai Zhang, Feng Li, Zhaohui Chen

**Affiliations:** 1 Department of Respiratory Medicine, Shanghai Chest Hospital, Shanghai Jiao Tong University, No.241, West Huaihai Road, Shanghai, 200030 China; 2 School of Mechanical and Electrical Engineering, Hunan City University, Yiyang, 413099 China; 3 College of Information Technology, Shanghai Jian Qiao University, Shanghai, 201306 China

**Keywords:** Pressure-controlled ventilation, Compliance, Airway resistance, Simulation, Chronic obstructive pulmonary disease

## Abstract

**Background:**

Assessing mechanical properties of the respiratory system (C_st_) during mechanical ventilation necessitates an end-inspiration flow of zero, which requires an end-inspiratory occlusion maneuver. This lung model study aimed to observe the effect of airflow obstruction on the accuracy of respiratory mechanical properties during pressure-controlled ventilation (PCV) by analyzing dynamic signals.

**Methods:**

A Hamilton C3 ventilator was attached to a lung simulator that mimics lung mechanics in healthy, acute respiratory distress syndrome (ARDS) and chronic obstructive pulmonary disease (COPD) models. PCV and volume-controlled ventilation (VCV) were applied with tidal volume (V_T_) values of 5.0, 7.0, and 10.0 ml/kg. Performance characteristics and respiratory mechanics were assessed and were calibrated by virtual extrapolation using expiratory time constant (RC_exp_).

**Results:**

During PCV ventilation, drive pressure (DP) was significantly increased in the ARDS model. Peak inspiratory flow (PIF) and peak expiratory flow (PEF) gradually declined with increasing severity of airflow obstruction, while DP, end-inspiration flow (EIF), and inspiratory cycling ratio (EIF/PIF%) increased. Similar estimated values of C_rs_ and airway resistance (R_aw_) during PCV and VCV ventilation were obtained in healthy adult and mild obstructive models, and the calculated errors did not exceed 5%. An underestimation of C_rs_ and an overestimation of R_aw_ were observed in the severe obstruction model.

**Conclusion:**

Using the modified dynamic signal analysis approach, respiratory system properties (C_rs_ and R_aw_) could be accurately estimated in patients with non-severe airflow obstruction in the PCV mode.

**Supplementary Information:**

The online version contains supplementary material available at 10.1186/s12890-024-03061-2.

## Background

Mechanical ventilation is an important lifesaving procedure with wide clinical applications for various critical conditions. The adequate setting of ventilator parameters should be based on the patient’s condition for optimal patient outcomes and to minimize ventilator-associated injury and complications [1, 2]. Pressure-controlled ventilation (PCV) is broadly used for cases of severe respiratory failure. PCV improves arterial oxygenation and decreases peak airway pressure because it decelerates inspiratory low. However, PCV has some limitations, including insufficient ventilation and excessive ventilation [3–8].

Currently, the dynamic properties of the respiratory system and engineering models for various diseases are exploited in the diagnosis and treatment of pulmonary disorders [9, 10]. However, mechanical features cannot be assessed directly during mechanical ventilation and are commonly presented as lumped indicators, including airway resistance (R_aw_) and compliance (C_rs_) [11, 12]. Static compliance (C_st_) is an important physiological index for evaluating the elastic properties of the overall respiratory system in invasive cases and is calculated by the ratio of the tidal volume to driving pressure [13]. C_st_ can be monitored by setting an end-inspiratory occlusion in the volume-controlled ventilation (VCV) mode. During PCV, an appropriate inspiratory time should be preset to acquire the approximate plateau pressure (P_plat_). Nevertheless, setting an appropriate inspiratory time may be challenging because the patient’s condition may change quickly. In addition, end-inspiration occlusion necessitates ventilation to be discontinued. Furthermore, this maneuver can be influenced by strong, spontaneous breathing. Therefore, other methods that do not require end-inspiratory occlusion need to be developed [14, 15].

Recently, methods have been proposed for the assessment of respiratory system properties without end-inspiration occlusion. Multiple linear regression (MLR), considering the least-squares fitting (LSF) technique, constitutes the most applied tool in recent years. It approximates C_st_ and R_aw_ with high accuracy in case of negligible spontaneous breathing effort [16–18]. Further tools encompass the constrained optimization strategy [19], electrical impedance tomography (EIT) monitoring, linear fitting of the flow velocity waveform, short expiratory occlusions, repeated changes in pressure support level, and artificial neural networks [20–23]. Still, the above techniques have some limitations: some do not adapt to spontaneous breathing conditions [16–18], while others apply sophisticated medical information or specific manual maneuvers, and others use empirical parameters [20, 22]. More importantly, most of them have more accurate measurements during VCV with constant inspiratory flow, with reduced accuracy when inspiratory flow is variable, such as in the PCV and PSV modes or spontaneous breathing effort. Secondly, the noise interference of ventilation waveforms exists in the real clinical setting, and noises encompass spontaneous breathing efforts, suctions, and coughing. Selecting adequate breaths that are less affected by noise might enhance accuracy in C_st_ and R_aw_ estimations.

In mechanically ventilated cases, expiration is a passive process depending on the expiratory time constant (RC_exp_) of the respiratory system. RC_exp_ reflects the mechanical features of the respiratory system [elastance and resistance (RC_exp_ = R_aw_×C_rs_)] and reveals the changes in the features of the pneumatic respiratory system [24]. C_rs_ and R_aw_ might be obtained from the passive deflation of lungs by using RC_exp_ and specific equations. In a previous bench study by the authors, the C_rs_ value was generally overestimated in the active breathing patient and underestimated in severe obstructive conditions, and the estimated error of R_aw_ by the RC_exp_ technique was minimal during passive breathing [15]. Recently, respiratory mechanics were estimated by modifying ventilation waveforms in the PCV mode to assess C_st_ and R_aw_ obtained by the end-inspiratory occlusion maneuver in the VCV mode. The continuous ventilation waveforms were examined, and an extra virtual tidal volume (V_T_) was calculated using RC_exp_ and an appropriate equation. Then, respiratory mechanics were estimated by analyzing the dynamic signals, which considerably improved static measurements. Such an approach improves estimation precision in respiratory system mechanics via real-time collection of respiratory data from the inspiration and expiration phases by applying specific Eqs. [25, 26]. C_st_ and R_aw_ measurements based on the end-inspiratory occlusion maneuver in the real clinical setting were considered the gold standard for the validation of the proposed approach. The present study aimed to assess the accuracy of respiratory mechanical properties by the extra virtual V_T_ in the PCV mode.

## Methods

### Lung models

The ASL 5000 Breathing Simulator (IngMar Medical, Pittsburg, PA, USA) features a computerized lung simulator with a piston that moves in a cylinder. This simulator was set to a single compartment based on a work by Beloncle et al. and previous bench studies by the authors [15, 27]. The applied respiratory mechanics parameters simulated an adult patient (65 to 70 kg body weight) placed in the semi-recumbent position. Six clinical scenarios with/without expiratory flow limitation (EFL) were constructed as follows [10, 28, 29]: healthy adult [inspiratory resistance (R_insp_) and expiratory resistance (R_exp_) of 5.0 cmH_2_O/L/s], mildly, moderate-to-severe obstruction [R_insp_=R_exp_=10.0, 15.0, and 20.0 cmH_2_O/L/s], severe obstruction with EFL [R_insp_=10.0 cmH_2_O/(L/s), R_exp_=20.0 cmH_2_O/L/s], and ARDS [R_insp_=R_exp_=10 cmH_2_O/L/s]. C_st_ was set at 30 (ARDS) and 60 (COPD) mL/cmH_2_O, and inspiratory time at 0.8 s (ARDS) and 1.6 s (COPD). Inspired oxygen fraction (F_I_O_2_) was 0.21 for all measurements.

### Ventilator settings

A dry circuit was used for the bench work, simulating a passive condition with both breathing frequency and P_mus_ of zero. A Hamilton C3 ventilator (Hamilton Medical AG, Bonaduz, Switzerland) was attached to the lung simulator calibrated by the end-inspiratory occlusion maneuver in the VCV mode utilizing a constant flow. The Hamilton C3 device was used in the VCV mode. Positive end-expiratory pressure (PEEP) was 5.0 cmH_2_O, and the backup breathing rate was 10 breaths/min. During VCV and PCV, respiratory mechanics setting was performed to maintain the output tidal volume (V_T_) at 5.0, 7.0, and 10.0 ml/kg. A reduced inspiratory rise time was applied to prevent overshooting in the PCV mode.

### Data collection

After baseline pressure stabilization, typical breaths were selected and recorded at 1-min intervals. Data were obtained for a total of six times after inspiratory pressure levels were adjusted in each lung model. All breaths were assessed offline using the ASL 5000 breathing simulator software.

Peak inspiratory flow (PIF), end-inspiratory flow (EIF), end-inspiratory pressure (EIP), and actual inspiratory time (T_I_) were determined using the simulator. Expiratory V_T_ was also evaluated. Peak expiratory flow (PEF) and total PEEP were collected in the expiration phase (Figure S1).

Respiratory mechanics indexes were considered the main determinants of the interaction between the patient and the ventilator. During PCV, the quasi-static two-point compliance of the respiratory system (C_rs_) was determined as V_T_ by driving pressure (DP). DP was the difference between EIP and total PEEP obtained at end-inspiration and end-expiration, respectively. RC_exp_ was the V_T_/flow ratio at 75% of expiratory V_T_ [30]. The equations representing these relationships are:

Extra virtual tidal volume:

1 $${{\rm{V}}_{{\rm{T}}\,{\rm{ virtual}}}}\,{\rm{ = }}\,{\rm{R}}{{\rm{C}}_{{\rm{exp}}}}{\rm{ \times EIF}}$$

2 $${{\rm{C}}_{{\rm{rs}}}}{\mkern 1mu} {\rm{ = }}{\mkern 1mu} \left( {{{\rm{V}}_{{\rm{TE}}}}{\rm{ + }}{{\rm{V}}_{{\rm{T}}\,{\rm{virtual}}}}} \right){\mkern 1mu} {\rm{/}}\left( {{\rm{EIP - PEEP}}} \right)$$

Inspiratory resistance (R_insp_) was derived from the following equations considering dynamic signals:

3 $${{\rm{P}}_{{\rm{Ers}}\,{\rm{insp}}}}{\mkern 1mu} {\rm{ = }}{\mkern 1mu} \left( {{{\rm{V}}_{{\rm{TE}}}}{\rm{ - }}{{\rm{V}}_{{\rm{PIF}}}}} \right){\rm{/}}{{\rm{C}}_{{\rm{rs}}}}$$

4 $${{\rm{R}}_{{\rm{insp}}}}{\mkern 1mu} {\rm{ = }}{\mkern 1mu} \left[ {{{\rm{P}}_{{\rm{PIF}}}}{\rm{ - }}\left( {{\rm{EIP - }}{{\rm{P}}_{{\rm{Ers}}\,{\rm{insp}}}}} \right)} \right]{\rm{/PIF}}$$

Expiratory resistance (R_exp_) was assessed with Eqs. 5 and 6:

5 $${{\rm{P}}_{{\rm{Ers}}\,{\rm{ exp}}}}\,{\rm{ = }}\,\left( {{{\rm{V}}_{{\rm{TE}}}}{\rm{ - }}{{\rm{V}}_{{\rm{PEF}}}}} \right){\rm{/}}{{\rm{C}}_{{\rm{rs}}}}$$

6 $${{\rm{R}}_{{\rm{exp}}}}{\mkern 1mu} {\rm{ = }}{\mkern 1mu} \left[ {{{\rm{P}}_{{\rm{PEF}}}}{\rm{ - }}\left( {{\rm{EIP - }}{{\rm{P}}_{{\rm{Ers}}\,{\rm{exp}}}}} \right)} \right]{\rm{/PEF}}$$

The percentages of measurement errors for compliance or resistance (%error C_rs_ and %error R_aw_) were calculated as follows [27]:

7 $${\rm{\% }}\,{\rm{error}}\,{\rm{ }}{{\rm{C}}_{{\rm{rs}}}}{\rm{ = }}\left( {{{\rm{C}}_{{\rm{rs - estimate}}}}{\rm{ - }}{{\rm{C}}_{{\rm{rs - VCV}}}}} \right){\rm{/}}{{\rm{C}}_{{\rm{rs - VCV}}}}{\rm{ \times 100\% }}$$

8 $${\rm{\% }}\,{\rm{error}}\,{\rm{ }}{{\rm{R}}_{{\rm{aw}}}}{\rm{ = }}\left( {{{\rm{R}}_{{\rm{aw - estimate}}}}{\rm{ - }}{{\rm{R}}_{{\rm{aw - VCV}}}}} \right){\rm{/}}{{\rm{R}}_{{\rm{aw - VCV}}}}{\rm{ \times 100\% }}$$

### Statistical analysis

All analyses were performed using SPSS 19.0 (IBM, Armonk, NY, USA). Data were shown as means ± standard deviations (SDs). The Shapiro-Wilk test was used for normality assessment. One-way ANOVA was used for comparisons in different settings. C_rs_, R_insp_, and R_exp_ were calculated in the VCV mode using the end-inspiration occlusion approach and the dynamic signal analysis method with extra virtual V_T_ in the PCV mode, with a two-tailed *t*-test for comparisons. Absolute differences between the extra virtual V_T_ and occlusion methods were determined, and *P* < 0.01 indicated statistical significance. Differences between PCV and VCV were determined as absolute percentages of values measured in the VCV mode.

## Results

### EIF and extra virtual V_T_ in the PCV mode under passive breathing

In PCV, EIF/PIF% was not above 5% in the non-severe obstructive lung models [R_aw_ ≤10.0 cmH_2_O/L/s] and close to 0 in the ARDS lung model. EIF/PIF% was increased with the aggravation of airflow obstruction, i.e., about 10% at a R_aw_ of 20.0 cmH_2_O/L/s (*P* < 0.001). Extra virtual V_T_ and the percentage of extra virtual V_T_ and V_TE_ (ΔV_T_%) were also increased (all *P* < 0.05). Compared with the normal adult lung model, there were significant differences in EIF/PIF% and ΔV_T_% under moderate to severe obstructive conditions (Table 1).

**Table 1:** EIF/PIF% and ΔV_T_% in various lung models in the PCV mode

	Normal adult (C_rs_=60, *R*_insp_=*R*_exp_=5.0)	Mild obstructive (C_rs_=60, *R*_insp_=*R*_exp_=10.0)	Moderate obstructive (C_rs_=60, *R*_insp_=*R*_exp_=15.0)	Severe obstructive (C_rs_=60, *R*_insp_=*R*_exp_=20.0)	Obstructive with EFL (C_rs_=60, *R*_insp_=10.0, *R*_exp_=20.0)	ARDS (C_rs_=30, *R*_insp_=*R*_exp_=10.0)
EIF/PIF%	1.34 ± 0.42	1.74 ± 0.86 (*t* = 1.7732) (*P* = 0.0426)	5.55 ± 0.65* (*t* = 23.0803) (*P* < 0.001)	10.86 ± 0.41* (*t* = 68.8143) (*P* < 0.001)	1.80 ± 0.48* (*t* = 3.0599) (*P* = 0.0022)	0.16 ± 0.20* (*t* = 10.7619) (*P* < 0.001)
ΔV_T_>%	1.12 ± 0.33	1.58 ± 0.75 (*t* = 2.3818) (*P* = 0.0115)	4.66 ± 0.61* (*t* = 21.6554) (*P* < 0.001)	10.07 ± 0.61* (*t* = 54.7503) (*P* < 0.001)	1.67 ± 0.77* (*t* = 2.7854) (*P* = 0.0043)	0.13 ± 0.16* (*t* = 11.4528) (*P* < 0.001)

*P value (Student’s t-test) for comparing normal adult and airflow obstruction lung models. Data are mean ± standard deviation, from 18 measurements/cases

### Estimation of C_rs_ in various models in the VCV and PCV modes

**Fig. 1:**
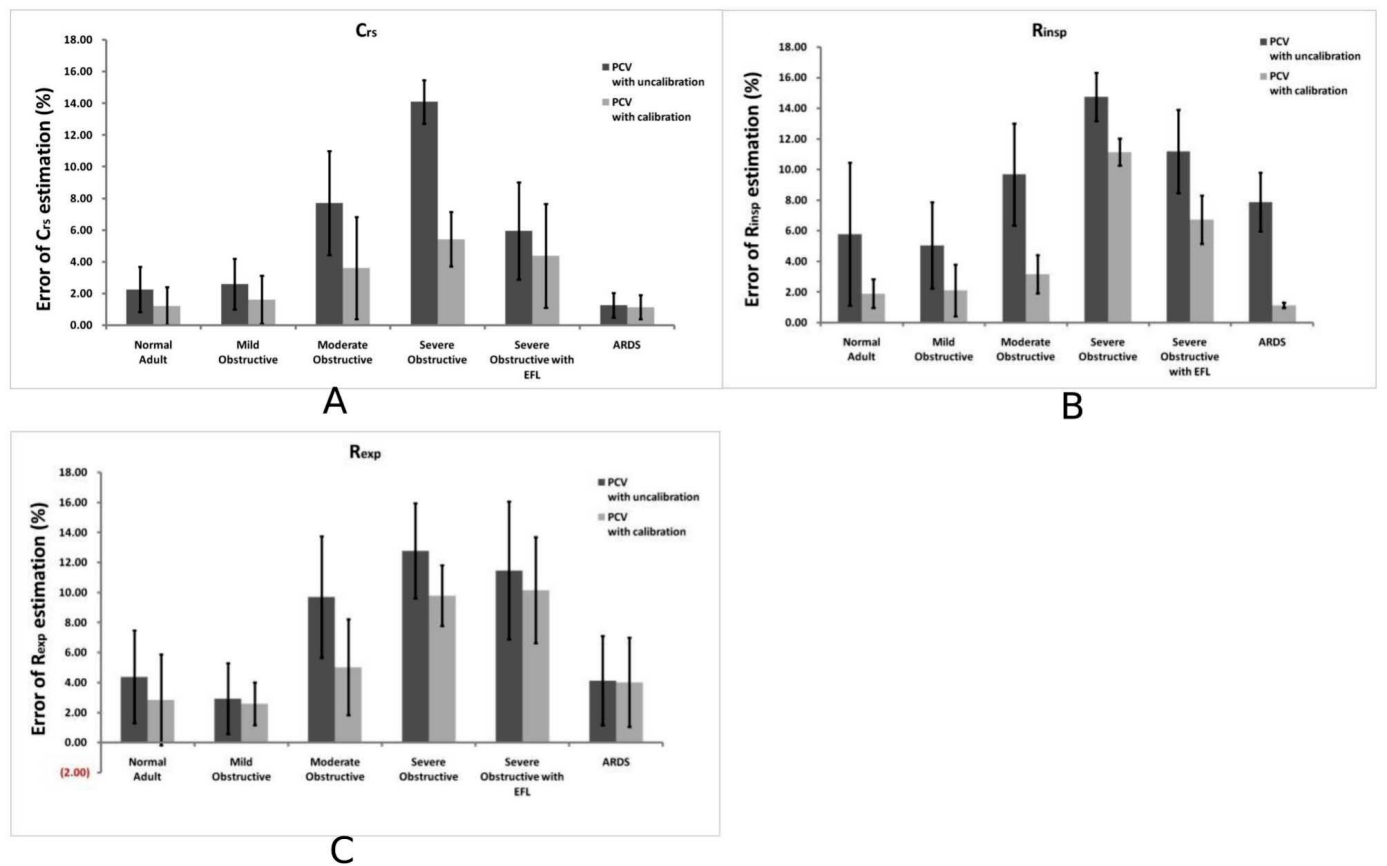
**(A)** Errors of system compliance (Crs) in various lung models during PC ventilation. **(B)** Errors of Rinsp in different lung models during PC ventilation. **(C)** Errors of Rexp in different lung models during PC ventilation. Data are shown as mean ± SD

Inspiratory V_T_ in the PCV mode was corrected by RC_exp_ and EIF. The estimated value of C_rs_ was larger than the value without extra virtual V_T_ calibration and close to the value obtained in the VCV mode with end-inspiratory occlusion. The estimated C_rs_ decreased significantly with increasing severity of airflow obstruction in either ventilatory mode, and uncalibrated C_rs_ values were only 49.28 ± 0.34 mL/cmH_2_O (PCV mode) and 57.38 ± 1.00 mL/cmH_2_O (VCV mode) (*P* < 0.01) in the severe obstructive lung model [R_aw_=20.0 cmH_2_O/L/s]. After the correction of extra virtual V_T_, the calculated errors were < 5% in all four lung models [R_aw_≤15.0 cmH_2_O/L/s], which showed no significant differences compared with estimated values in the VCV mode (Table 2; Fig. 1A).

**Table 2:** System compliance (C_rs_) among lung models in different ventilatory modes

	Normal adult (C_rs_=60, *R*_insp_=*R*_exp_=5.0)	Mild obstructive (C_rs_=60, *R*_insp_=*R*_exp_=10.0)	Moderate obstructive (C_rs_=60, *R*_insp_=*R*_exp_=15.0)	Severe obstructive (C_rs_=60, *R*_insp_=*R*_exp_=20.0)	Obstructive with EFL (C_rs_=60, *R*_insp_=10.0, *R*_exp_=20.0)	ARDS (C_rs_=30, *R*_insp_=*R*_exp_=10.0)
VCV	60.37 ± 0.65	59.93 ± 0.74	59.24 ± 2.06	57.38 ± 1.00	56.17 ± 1.23	30.16 ± 0.24
PCV	58.99 ± 0.60	58.37 ± 0.83	54.62 ± 1.15	49.28 ± 0.34	52.79 ± 1.02	29.97 ± 0.20
PCV-cal	59.92 ± 0.66* (*t* = 2.0610) (*P* = 0.047)	59.03 ± 0.84	57.16 ± 1.25	54.24 ± 0.30	53.67 ± 1.04	30.01 ± 0.19* (*t* = 2.0790) (*P* = 0.045)
*F*	21.96	17.05	40.56	747.05	45.68	18.59
*P*	< 0.01	< 0.01	< 0.01	< 0.01	< 0.01	< 0.01

## Estimation of R_aw_ in various models in the VCV and PCV modes

There were similar estimated R_insp_ and R_exp_ in the PCV and VCV modes with R_aw_ ≤10.0 cmH_2_O/L/s, and calculated errors were ≤ 10.0%. After V_T_ calibration, the estimated errors of R_insp_ and R_exp_ were reduced to < 5%. In severe obstructive and obstructive with EFL models, the estimated errors of R_insp_ and R_exp_ were significantly reduced and were below 10% after V_T_ calibration (Tables 3 and 4; Fig. 1B and C).

**Table 3:** Comparison of R_insp_ between lung models in different ventilatory modes

	Normal adult (C_rs_=60, *R*_insp_=*R*_exp_=5.0)	Mild obstructive (C_rs_=60, *R*_insp_=*R*_exp_=10.0)	Moderate obstructive (C_rs_=60, *R*_insp_=*R*_exp_=15.0)	Severe obstructive (C_rs_=60, *R*_insp_=*R*_exp_=20.0)	Obstructive with EFL (C_rs_=60, *R*_insp_=10.0, *R*_exp_=20.0)	ARDS (C_rs_=30, *R*_insp_=*R*_exp_=10.0)
VCV	5.05 ± 0.28	10.04 ± 0.25	14.93 ± 0.41	19.68 ± 0.37	10.25 ± 0.38	10.54 ± 1.02
PCV	5.27 ± 0.15	10.53 ± 0.39	16.31 ± 0.39	22.57 ± 0.81	11.38 ± 0.32	10.10 ± 0.33
PCV-cal	5.18 ± 0.14* (*t* = 1.7618) (*P* = 0.087)	10.72 ± 0.51	15.78 ± 0.43	22.38 ± 0.71	12.07 ± 0.43	10.09 ± 0.34* (*t* = 1.7757) (*P* = 0.085)
*F*	5.48	14.00	51.81	108.78	105.60	2.82
*P*	0.007	< 0.01	< 0.01	< 0.01	< 0.01	0.069

*P-values (Student t-test) are for comparisons between the VCV and PCV modes. Data are shown as means ± standard deviations and are the results of 18 measurements/cases

**Table 4:** R_exp_ values in lung models in different ventilatory modes

	Normal adult (C_rs_=60, *R*_insp_=*R*_exp_=5.0)	Mild obstructive (C_rs_=60, *R*_insp_=*R*_exp_=10.0)	Moderate obstructive (C_rs_=60, *R*_insp_=*R*_exp_=15.0)	Severe obstructive (C_rs_=60, *R*_insp_=*R*_exp_=20.0)	Obstructive with EFL (C_rs_=60, *R*_insp_=10.0, *R*_exp_=20.0)	ARDS (C_rs_=30, *R*_insp_=*R*_exp_=10.0)
VCV	5.38 ± 0.18	10.35 ± 0.22	14.69 ± 0.77	19.93 ± 0.47	19.44 ± 1.13	10.18 ± 0.29
PCV	5.57 ± 0.13	10.59 ± 0.59	16.08 ± 0.31	22.47 ± 0.41	20.48 ± 0.84	10.48 ± 0.28
PCV-cal	5.41 ± 0.15* (*t* = 0.5432) (*P* = 0.587)	10.42 ± 0.29* (*t* = 0.8159) (*P* = 0.420)	15.12 ± 0.26* (*t* = 2.2448) (*P* = 0.0314)	21.77 ± 0.36	20.06 ± 0.80* (*t* = 1.8999) (*P* = 0.066)	10.36 ± 0.28* (*t* = 1.8944) (*P* = 0.0667)
*F*	7.85	1.71	36.15	179.22	5.64	5.11
*P*	0.0011	0.1908	< 0.01	< 0.01	0.0061	0.0095

*P values (Student’s t-test) are for comparisons between the VCV and PCV. Data are mean ± standard deviation from 18 measurements/cases

### Bland-Altman analysis of differences between the PCV and VCV modes

**Fig. 2:**
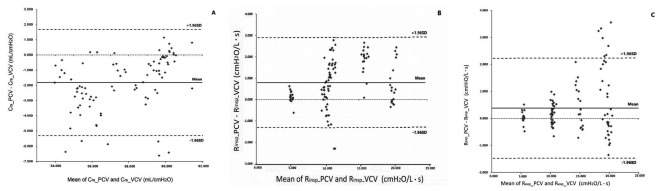
Bland-Altman plots depicting system compliance (**A**), inspiratory resistance (**B**) and expiratory resistance (**C**) by the V_T_ calibration and end-inspiration occlusion approaches. Data from 5 (C_rs_) and 6 (R_aw_) lung models, in totally 168 breaths. Each circle reflects one breath for a given model. Dashed lines in the middle depict mean differences between the V_T_ calibration and end-inspiration occlusion approaches. The remaining two dashed lines are mean ± 1.96*SD

**Fig. 3:**
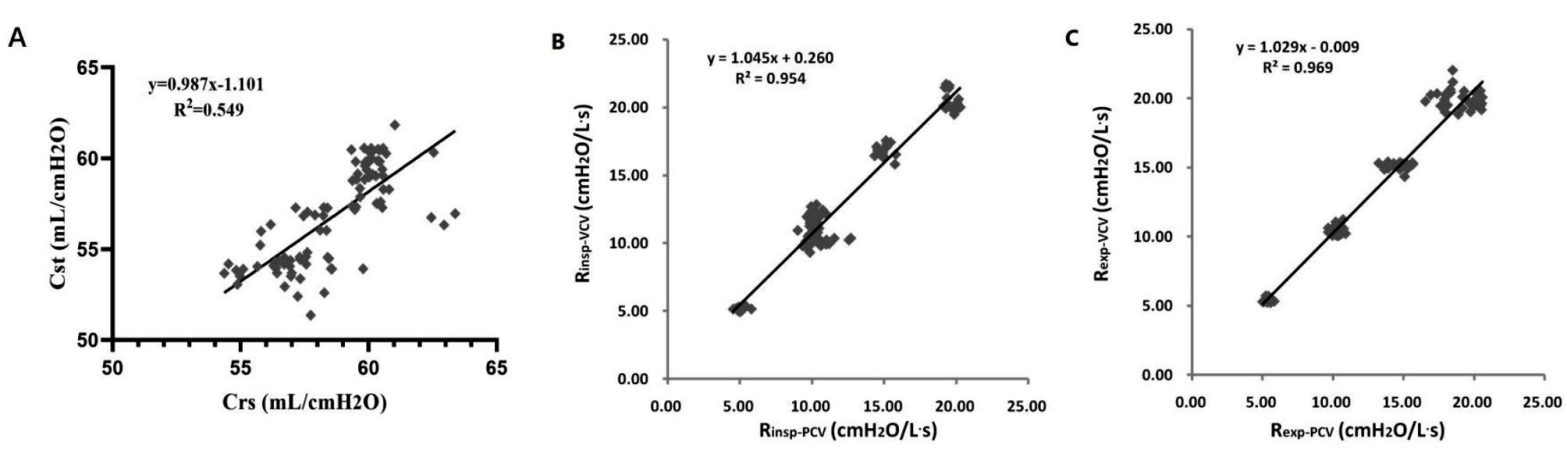
Associations of estimated C_rs_ (**A**), R_insp_ (**B**) and R_exp_ (**C**) with gold standard obtained by the end-inspiration occlusion approach. Data from 5 (C_rs_) and 6 (R_aw_) lung models, totally 168 breaths were examined. Each circle reflects one breath for a given model

In all five lung profiles with normal system compliance (60.0 mL/cmH_2_O), the difference in C_rs_ between the V_T_ calibration and end-inspiration occlusion approaches was 1.82 *±* 1.43 mL/cmH_2_O; the weighted correlation coefficient of C_rs_ equaled 0.549 after V_T_ calibration (*P* < 0.001). The differences of R_insp_ and R_exp_ values in all lung models were 0.79 ± 1.96 × 0.89 and 0.38 ± 1.96 × 0.69 cmH_2_O/L/s, and the weighted correlation coefficients of R_insp_ and R_exp_ were equal to 0.954 and 0.969, respectively (all *P* < 0.001) (Figs. 2 and 3).

## Discussion

This bench study mostly revealed the following. (1) During PCV under the passive breathing condition, the estimated error was affected by the severity of airway obstruction, significantly underestimated in C_rs_, and significantly overestimated in R_aw_ without V_T_ calibration. (2) In the non-severe obstructive condition [R_aw_ ≤ 10.0 cmH_2_O/L/s], estimated errors were ≤ 10% in calculated C_rs_ and R_aw_. (3) The estimated accuracies of C_rs_, R_insp_, and R_exp_ were improved by V_T_ calibration with extra virtual inspiratory volume.

During mechanical ventilation, assessing the respiratory mechanics by end-inspiratory occlusion with a constant inspiratory flow is a classic measurement method. However, the occlusion technique may be performed with no gas flow and fixed tidal volume. It is important for the patient to make no efforts during static measurements, whether related to disease, sedation, or paralysis, and special ventilatory settings are also required (such as constant inspiratory flow and end-inspiration pause) [31–33]. The most important concern is that measurement data are reflected by mechanical properties under static or quasi-static conditions. The occlusion method could neither be adapted to the PCV mode since inspiratory flow is always variable nor be used in assisted ventilation in which the spontaneous effort is always present and variable. Recently, several continuous respiratory mechanics measurement techniques, including LSF and expiratory time constant method (RC_exp_), have been developed. These newer approaches not only have good adaptability and anti-noise-interference performance but also could be applied during assisted mechanical ventilation with spontaneous breathing [34].

Volta et al. found that EFL substantially reduces the accuracy of resistance and compliance assessed by the LSF method; the determination of respiratory indexes during inspiration helps evaluate respiratory mechanics in flow-limited COPD cases, and the LSF technique could detect PEEPi_dyn_ only using inspiratory data [18]. However, the estimated error of LSF was affected by the spontaneous breathing effort, and Raw underestimation and C_rs_ overestimation were observed in the PSV mode [16, 35]. The other approaches have certain limitations. Some could not deal with significant spontaneous breathing, while others are based on sophisticated medical equipment or manual maneuvers, preventing their routine clinical use [18–22]. Recently, Pan et al. proposed a tool measuring quasi-static respiratory system compliance (C_q−stat_) in the PCV mode without the need for the end-inspiratory occlusion maneuver, with a virtual assessment of flow-time waveforms with end-inspiration flow not equaling zero, to allow for C_q−stat_ determination [14]. In this bench study, the dynamic signal analysis approach was used to collect and calculate gas flow, airway pressure, and volume data at different time points during mechanical ventilation, and the estimated C_rs_, R_insp_, and R_exp_ were calibrated by virtual extrapolation of V_T_ when end-inspiration flow was not zero. Dynamic signal analysis does not require special maneuvers such as long-time pauses at the inspiration or expiration phase [26]. In healthy adults and mild obstruction lung models, no significant differences were found in estimated C_rs_ and R_aw_ (R_insp_ and R_exp_) between the PCV and VCV modes with EIF < 2.0 L/min and EIF/PIF% < 5.0%. The calculated error was increased when EIF/PIF% was above 5% in the PCV mode. Due to the exacerbation of airflow obstruction, PIF was decreased, and EIF did not drop to zero at the end of inspiration. EIF/PIF% was increased to about 10%, resulting in V_T_ decrease, C_rs_ underestimation, and R_aw_ overestimation. After V_T_ calibration with RC_exp_ and EIF, the accuracy of the estimation was improved significantly, and the values obtained were similar to those estimated in the VCV mode by the occlusion method.

In the classic system compliance (C_rs_) calculation equation, C_rs_ is the ratio of the monitored tidal volume (V_T_) to the driving pressure (DP) of the airway. The tidal volume is the sum of the gas output capacity of the ventilator during the inhalation phase. In the classic calculation scheme, the tidal volume is the gas output capacity value measured after the end-inspiratory flow rate reaches 0. On the other hand, in this study, due to the special nature of the PCV mode, the end-inspiratory flow rate does not always decrease to 0. Therefore, an additional parameter (i.e., the extra virtual tidal volume) was designed to simulate the gas capacity generated after the flow rate continued to decrease to 0. It was found that the addition of the extra virtual tidal volume was of great significance for calculating C_rs_ under the PCV ventilation state.

One of the limitations of the present bench study is the standardization of simulation indexes for the respiratory system’s mechanics in the lung model. Although the mechanical lung simulator cannot completely replace animal experiments and real clinical practice, the ASL 5000 mechanical lung simulator also has its unique advantages. Firstly, it can simulate simple single-chamber linear models and complex lung mechanics models with dual-chamber nonlinearity. In this study, we attempted to explore a new respiratory mechanics calculation scheme that can be applied to non-interrupted breathing and non-constant inspiratory flow mechanical ventilation conditions and can accurately calculate the respiratory mechanics characteristics of patients with different respiratory system diseases. Therefore, a mechanical lung simulator was first used for the experiment because its output data is relatively stable, and this lung simulator is also often selected in many mechanical simulation experiments. Further animal experiments will be conducted in the future [36]. Secondly, the passive breathing condition was simulated during PCV since spontaneous breathing effort might affect V_T_ and C_rs_. It is not clear whether this scheme could be applied to other assisted ventilatory modes such as PSV. Thirdly, airway resistance varies with gas flow through the trachea and bronchus. Therefore, the above data reflect maximal resistance in a patient during breathing, and whether they can be translated in the clinical setting is unknown. Therefore, further clinical trials are warranted.

## Conclusion

Using the modified dynamic signal analysis approach, respiratory system properties (C_rs_ and R_aw_) could be accurately estimated in patients with non-severe airflow obstruction in the PCV mode. Compared with the VCV mode with constant flow, inspiratory flow decreased exponentially in the PCV mode. PIF and the deceleration rate of inspiratory flow were dependent upon the mechanical characteristics of the respiratory system, especially airflow obstruction.

### Electronic supplementary material

Below is the link to the electronic supplementary material.

Supplementary Material 1

Supplementary Material 2

## Data Availability

All data generated or analyzed during this study are included in this article.

## Abbreviations

PCV: Pressure-controlled ventilation
COPD: Chronic obstructive pulmonary disease
VCV: Volume-controlled ventilation
V_T_: Tidal volume
DP: Drive pressure
PIF: Peak inspiratory flow
PEF: Peak expiratory flow
EIF: End-inspiration flow
EIF/PIF%: Inspiratory cycling ratio
R_aw_: Airway resistance
C_st_: Static compliance
P_plat_: Plateau pressure
MLR: Multiple linear regression
LSF: Least-squares fitting
EIT: Electrical impedance tomography
RC_exp_: Expiratory time constant
EFL: Expiratory flow limitation
PEEP: Positive end-expiratory pressure
EIP: End-inspiration pressure
T_I_: Inspiratory time
C_rs_: Respiratory system compliance
DP: Driving pressure
SD: Standard deviation
C_q−stat_: Quasi-static respiratory system compliance
