# Supplementary figures and images for: Continuous estimation of respiratory system compliance and airway resistance during pressure-controlled ventilation without end-inspiration occlusion
Source: BMC Pulm Med. 2024 May 20;24:249. doi: 10.1186/s12890-024-03061-2 (PMC11107031; doi:10.1186/s12890-024-03061-2)

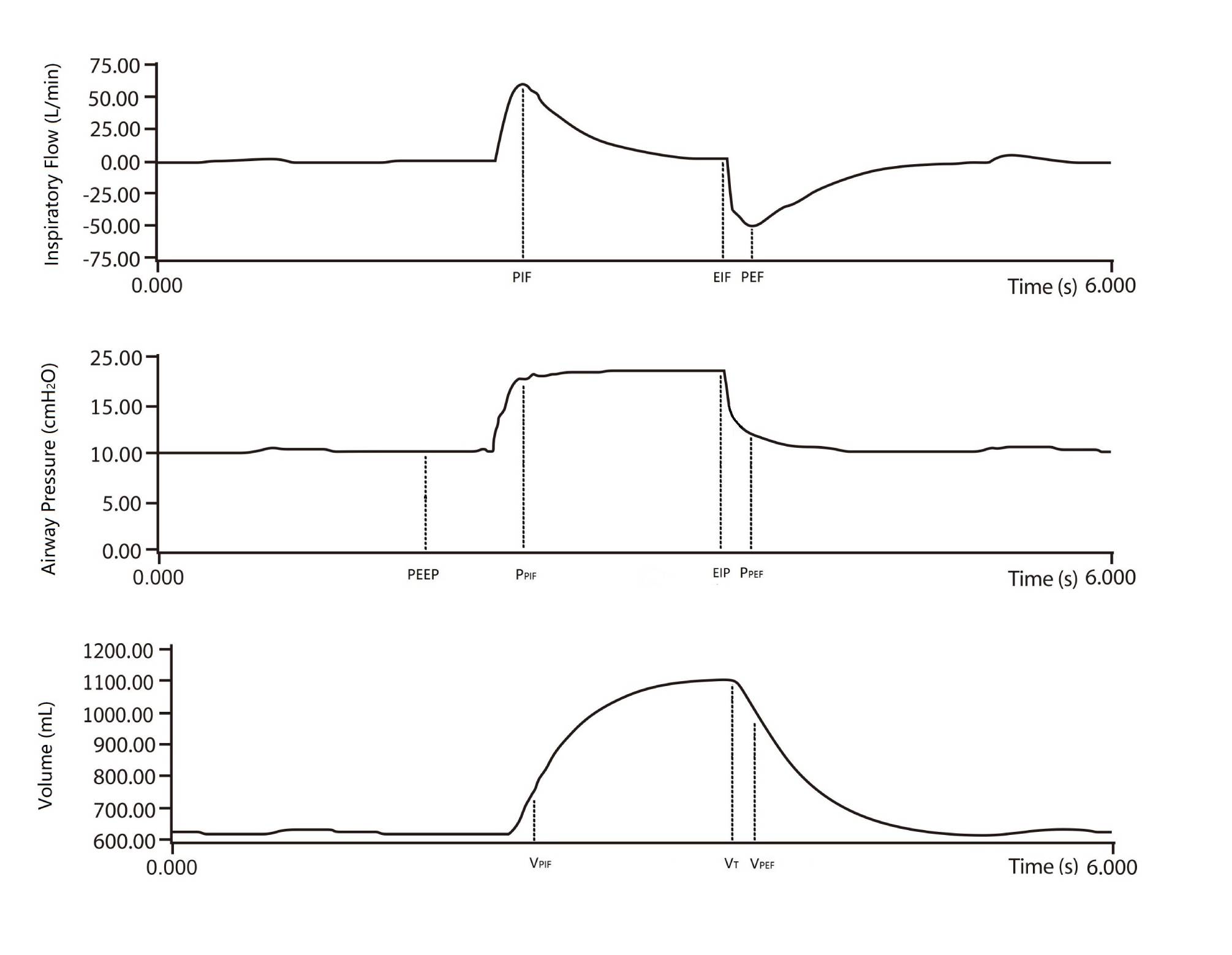

Supplement: Supplementary file 1 — Supplementary Material 1 [file 12890_2024_3061_MOESM1_ESM.jpg]
