# Supplementary material for: Continuous estimation of respiratory system compliance and airway resistance during pressure-controlled ventilation without end-inspiration occlusion
Source: BMC Pulm Med. 2024 May 20;24:249. doi: 10.1186/s12890-024-03061-2 (PMC11107031; doi:10.1186/s12890-024-03061-2)
Supplement: Supplementary file 2 — Supplementary Material 2 [file 12890_2024_3061_MOESM2_ESM.docx]

**Figure S1.** Airway pressure, flow, and volume waveforms during pressure controlled ventilation (PCV), illustrating the characteristics of pressure-controlled breathing. With a period of exponential decay flow, the end-inspiration airway pressure is maintained as the plateau (EIP). Tidal volume (V_T_) is determined by the difference between EIP and PEEP and respiratory system compliance.
